# RETRACTED: Abou-ElNaga et al. Novel Nano-Therapeutic Approach Actively Targets Human Ovarian Cancer Stem Cells After Xenograft into Nude Mice. *Int. J. Mol. Sci.* 2017, *18*, 813

**DOI:** 10.3390/ijms27209309

**Published:** 2026-10-20

**Authors:** Amoura Abou-ElNaga, Ghada Mutawa, Ibrahim M. El-Sherbiny, Hassan Abd-ElGhaffar, Ahmed A. Allam, Jamaan Ajarem, Shaker A. Mousa

**Affiliations:** 1 Zoology Department, Faculty of Sciences, Mansoura University, Mansoura 35516, Egypt; dramabouelnaga@yahoo.com (A.A.-E.); ghada.adel.mutawa@gmail.com (G.M.); 2 Center for Materials Science, Zewail City of Science and Technology, Cairo 12588, Egypt; ielsherbiny@zewailcity.edu.eg; 3 Clinical Pathology Department, Faculty of Medicine, Mansoura University, Mansoura 35516, Egypt; haabdelghaffar@yahoo.com; 4 Zoology Department, College of Science, King Saud University, Riyadh 11451, Saudi Arabia; aallam@ksu.edu.sa (A.A.A.); jajarem@ksu.edu.sa (J.A.); 5 Zoology Department, Faculty of Science, Beni-Suef University, Beni-Suef 62511, Egypt; 6 The Pharmaceutical Research Institute, Albany College of Pharmacy and Health Sciences, Rensselaer, NY 12144, USA

The journal retracts the article titled “Novel Nano-Therapeutic Approach Actively Targets Human Ovarian Cancer Stem Cells after Xenograft into Nude Mice” [1], cited above.

Following the publication of a correction [2] to this article, further concerns were raised regarding the integrity of the images presented in this publication.

In accordance with the journal’s standard procedures, the Editorial Office and Editorial Board conducted an investigation that identified additional indications of inappropriate image editing in Figure 5b of the corrected article [2]. Although the authors cooperated with the investigation, the explanations and supporting materials provided were not deemed sufficient by the Editorial Board to resolve the identified concerns.

As a result, the Editorial Board has lost confidence in the reliability of the findings reported in this article [1] and has decided to retract the publication in accordance with MDPI’s retraction policy.

This retraction was approved by the Editor-in-Chief of the *International Journal of Molecular Sciences*.

The authors have been informed of this retraction and have indicated their disagreement with this decision.
